# Supplementary material for: Dynamics of sex-biased gene expression during development in the stick insect Timema californicum
Source: Heredity (Edinb). 2022 May 17;129(2):113–22. doi: 10.1038/s41437-022-00536-y (PMC9338061; doi:10.1038/s41437-022-00536-y)
Supplement: Supplementary file 1 — Supplemental material [file 41437_2022_536_MOESM1_ESM.pdf]

Supplemental material

Supplemental table 1| The sex of *Timema* hatchlings was determined via X-linked microsatellite genotyping using the indicated primer sequences **(a)** and PCR conditions **(b)**

a)

Usat ID	Primer ID	Forward 5'-3'	Primer ID	Reverse 5'-3'
**19**	Cm_19F	CATCGAGTGTTGCTAAGAATGG	Cm_19R	AGACTTTAACCCAGGCCCAG
**20**	Cm_20F	TGTGCGAGCAAATGAGGTTG	Cm_20R	CGATGGCATGTCTTAAGCCC
**21**	Cm_21F	GCCAATCGAAGCGGGTATTG	Cm_21R	GTATGGAGCGAGTGACAAAGC
**24**	Cm_24F	TCGCTGCACATTTCTACAAAC	Cm_24R	CTGAATACCGGCAGTGAAGG

b)

**Cycling conditions**			
	**Temp**	**Time**	**Cycles**
**Denaturation initial**	95°C	15 min	
**Denaturation**	94°C	30sec	
**Annealing**	57°C	90sec	35x
**Extension**	72°C	1 min	
**Final extension**	60°C	30min	
	4°C	10 min	
	12°C	xxx	

Supplemental table 2| Genotypes for four X-linked microsatellite loci (Marker: 19, 20, 21 and 24) recorded in 15 male and 15 female adults and nine hatchlings. Heterozygotes are highlighted in grey.

Stage	Sample ID	Sex	Marker 19		Marker 20		Marker 21		Marker 24	
**Adult**	HM217	F	376	386	390	390	281	312	197	219
	HM218	F	351	383	390	390	277	312	200	200
	HM219	F	353	370	394	394	281	308	197	203
	HM220	F	353	390	390	394	277	308	191	191
	HM221	F	374	403	394	400	304	312	191	197
	HM222	F	388	395	396	412	277	308	197	206
	ReSeq_Cm02	F	351	351	390	398	288	308	203	203

	ReSeq_Cm04	F	353	374	392	394	292	312	191	197
	ReSeq_Cm06	F	374	393	390	394	277	281	197	197
	ReSeq_Cm08	F	353	392	396	396	296	304	197	197
	ReSeq_Cm10	F	351	360	392	394	308	312	203	203
	ReSeq_Cm12	F	351	393	394	394	277	312	197	206
	ReSeq_Cm14	F	355	355	394	394	288	308	191	203
	ReSeq_Cm16	F	370	378	394	396	304	304	197	200
	ReSeq_Cm18	F	378	390	394	396	277	292	197	197
	HM157	M	351		392		304		197	
	HM157b	M	369		390		274		197	
	HM158	M	380		394		281		197	
	HM159	M	348		392		324		194	
	HM160	M	386		394		292		206	
	HM161	M	376		394		292		203	
	HM162	M	393		396		308		NA	
	HM163	M	351		390		304		NA	
	HM164	M	351		394		308		197	
	HM165	M	382		394		304		201	
	HM166	M	376		392		292		200	
	Tcm_M_Hm01	M	374		396		308		206	
	Tcm_M_Hm02	M	390		410		324		197	
	Tcm_M_Hm03	M	351		408		296		195	
	Tcm_M_Hm04	M	NA		NA		304		NA	
**Hatchling**	Tim_Ha09	F	353	356	399		296		196	199
	Tim_Ha10	M	353		405		NA		196	
	Tim_Ha11	M	353		399		NA		199	
	Tim_Ha14	M	355		397		NA		199	
	Tim_Ha15	F	351	355	388	397	NA		196	
	Tim_Ha16	M	353		399		NA		199	
	TIM_HA12	F	353	356	399	405	295		NA	
	TIM_HA13	M	353		388		299		NA	
	TIM_HA18	F	353	356	NA	NA	295		NA	

Supplemental table 3| – *D. melanogaster* samples, and their accession numbers

**Accession**	**Sample ID**	**Sex**	**Stage**
SRR3092025	FL.H169.1	Female	Larva
SRR3091999	ML.H8.1	Male	Larva
SRR3092040	FL.H60.3	Female	Larva

SRR3092044	FL.H74.3	Female	Larva
SRR3091997	ML.H75.2	Male	Larva
SRR3091988	ML.H55.3	Male	Larva
SRR3092052	FL.H94.2	Female	Larva
SRR3091990	ML.H60.2	Male	Larva
SRR3092081	FP.H8.2	Female	Pupa
SRR3092085	FP.H94.3	Female	Pupa
SRR3092079	FP.H75.3	Female	Pupa
SRR3138724	MP.H163.2	Male	Pupa
SRR3138731	MP.H184.3	Male	Pupa
SRR3092055	FP.H163.1	Female	Pupa
SRR3138728	MP.H169.3	Male	Pupa
SRR3138734	MP.H196.3	Male	Pupa
SRR3092076	FA.H184.3	Female	Adult
SRR3092017	FA.H8.3	Female	Adult
SRR3091979	MA.H94.1	Male	Adult
SRR3091965	MA.H55.3	Male	Adult
SRR3092011	FA.H74.3	Female	Adult
SRR3091970	MA.H74.2	Male	Adult
SRR3091994	FA.H55.2	Female	Adult
SRR3091973	MA.H75.1	Male	Adult

Supplemental table 4|- *Timema californicum*; Filtered genes (Number of genes filtered for low expression), kept (genes included for differential gene expression analysis), Fb (female-biased genes), Mb (male-biased genes), total SBG (sum of the male and female-biased genes), %SBG (percentage of sex-biased genes), % (>1 log_2FC) (percentage of genes with strong sex bias). Genes are classified based on their sex-bias into seven categories: “slight FB”- female bias (<1 log_2FC), “strong FB”- female bias (≥1 log_2FC), “female limited”- with no expression in males, “slight MB”- male bias (<1 log_2FC), “strong MB”- male bias (≥1 log_2FC), “male limited”- no expression in females, “Not DE”- not differentially expressed genes.

	Filtered genes	Kept	Fb	Mb	total SBG	%SBG	% (>1 log2FC)	Slight FB	Slight MB	Strong FB	Strong MB	Female limited	Male limited	NotDE
hatchling	2763	11800	4	22	26	0.22	0.22	0	0	22	4	0	0	11774
juvenile	2469	12094	29	539	568	4.70	4.69	1	0	27	417	1	122	11526
adult	2313	12250	1023	1462	2485	20.29	15.60	374	200	646	1139	3	123	9765

Supplemental table 5| - *Drosophila melanogaster*; Filtered genes (Number of genes filtered for low expression), kept (genes included for differential gene expression analysis), Fb (female-biased genes), Mb (male-biased genes), total SBG (sum of the male and female-biased genes), %SBG (percentage of sex-biased genes), % (>1 $\log_2$FC) (percentage of genes with strong sex bias). Genes are classified based on their sex-bias into seven categories: “slight FB”- female bias (<1 $\log_2$FC), “strong FB”- female bias (≥1 $\log_2$FC), “female limited”- with no expression in males, “slight MB”- male bias (<1 $\log_2$FC), “strong MB”- male bias (≥1 $\log_2$FC), “male limited”- no expression in females, “Not DE”- not differentially expressed genes.

	Filtered genes	Kept	Fb	Mb	total SBG	%SBG	%(>1 log2FC)	Slight FB	Slight MB	Strong FB	Strong MB	Female limited	Male limited	NotDE
larva	6733	11023	399	1714	2113	19.17	15.81	202	168	197	1301	0	245	8910
pupa	5578	12178	151	2492	2643	21.70	20.41	63	94	88	1620	0	778	9357
adult	5283	12473	4497	5982	10479	84.00	68.91	1299	585	3181	4300	17	1097	1994

Supplemental table 6| Statistical results: partial correlations.

Female-biased adult stage

n=443 gp=2	dNdS	avr. exp.	GC	|FC|
**Estimate**				
dNdS	1.00	-0.03	-0.26	0.32
avr. exp	-0.03	1.00	0.03	-0.19
GC	-0.26	0.03	1.00	0.27
|FC|	0.32	-0.19	0.27	1.00
**p value**	dNdS	avr. exp.	GC	|FC|
dNdS	0.000	0.538	0.000	0.000
avr. exp	0.538	0.000	0.533	0.000
GC	0.000	0.533	0.000	0.000
|FC|	0.000	0.000	0.000	0.000
**statistic**	dNdS	avr. exp.	GC	|FC|
dNdS	0.00	-0.62	-5.70	7.06
avr. exp	-0.62	0.00	0.62	-4.13
GC	-5.70	0.62	0.00	5.95
|FC|	7.06	-4.13	5.95	0.00

Male-biased- adult stage

n=388 gp=2	dNdS	avr. exp.	GC	|FC|
**Estimate**				
dNdS	1.00	0.00	-0.44	0.19
avr. exp	0.00	1.00	0.08	-0.58
GC	-0.44	0.08	1.00	0.22
|FC|	0.19	-0.58	0.22	1.00
**p value**	dNdS	avr. exp.	GC	|FC|
dNdS	0.000	0.975	0.000	0.000
avr. exp	0.975	0.000	0.099	0.000
GC	0.000	0.099	0.000	0.000
|FC|	0.000	0.000	0.000	0.000
**statistic**	dNdS	avr. exp.	GC	|FC|
dNdS	0.00	0.03	-9.70	3.81
avr. exp	0.03	0.00	1.65	-14.06
GC	-9.70	1.65	0.00	4.42
|FC|	3.81	-14.06	4.42	0.00

Female-biased juvenile

n=11 gp=2	dNdS	avr. exp.	GC	|FC|
**Estimate**				
dNdS	1.00	-0.03	-0.07	0.40
avr. exp	-0.03	1.00	0.01	-0.44
GC	-0.07	0.01	1.00	-0.40
|FC|	0.40	-0.44	-0.40	1.00
**p value**	dNdS	avr. exp.	GC	|FC|
dNdS	0.000	0.932	0.932	0.573
avr. exp	0.932	0.000	0.974	0.240
GC	0.850	0.974	0.000	0.289
|FC|	0.286	0.240	0.289	0.000
**statistic**	dNdS	avr. exp.	GC	|FC|
dNdS	0.00	-0.09	-0.20	1.15
avr. exp	-0.09	0.00	0.03	-1.28
GC	-0.20	0.03	0.00	-1.15
|FC|	1.15	-1.28	-1.15	0.00

Male-biased juvenile

n= 94 gp=2	dNdS	avr. exp.	GC	|FC|
**Estimate**				
dNdS	1.00	-0.10	-0.34	0.08
avr. exp	-0.10	1.00	-0.08	-0.38
GC	-0.34	-0.08	1.00	-0.07
|FC|	0.08	-0.38	-0.07	1.00
**p value**	dNdS	avr. exp.	GC	|FC|
dNdS	0.000	0.433	0.002	0.452
avr. exp	0.325	0.000	0.457	0.000
GC	0.001	0.457	0.000	0.491
|FC|	0.452	0.000	0.491	0.000
**statistic**	dNdS	avr. exp.	GC	|FC|
dNdS	0.00	-0.99	-3.41	0.76
avr. exp	-0.99	0.00	-0.75	-3.93
GC	-3.41	-0.75	0.00	-0.69
|FC|	0.76	-3.93	-0.69	0.00

Female-biased hatchling

n=14 gp=2	dNdS	avr. exp.	GC	|FC|
**Estimate**				
dNdS	1.00	0.50	-0.52	0.51
avr. exp	0.50	1.00	0.11	-0.05
avr. exp. females	-0.52	0.11	1.00	0.33
|log2FC|	0.51	-0.05	0.33	1.00
**p value**	dNdS	avr. exp.	GC	|FC|
dNdS	0.000	0.174	0.151	0.162
avr. exp	0.174	0.000	0.777	0.905
GC	0.151	0.777	0.000	0.386
|FC|	0.162	0.905	0.386	0.000
**statistic**	dNdS	avr. exp.	GC	|FC|
dNdS	0.00	1.51	-1.61	1.56
avr. exp	1.51	0.00	0.29	-0.12
GC	-1.61	0.29	0.00	0.92
|FC|	1.56	-0.12	0.92	0.00

Supplemental table 7| Exact test of multi set intersections of sex-biased genes between developmental stages in *T. californicum*

Intersections	Degree	Observed Overlap	Expected Overlap	FE	P.value	P.adj
Adult	1	2485	NA	NA	NA	NA
Juvenile	1	568	NA	NA	NA	NA
Hatch	1	26	NA	NA	NA	NA
Juvenile & Adult	2	390	106.74	3.65	2.89E-157	1.16E-156
Hatch & Adult	2	12	4.89	2.46	0.001	1.32E-03
Hatch & Juvenile	2	15	1.12	13.43	1.30E-14	2.60E-14
Hatch & Juvenile & Adult	3	9	0.21	42.88	3.75E-13	5.00E-13

Supplemental table 8| Number of sex-biased genes (SB) overlapping between stages (SB in № stages), showing a significant sex by developmental stage interaction (Sex by development interaction) in *T. californicum*.

SB in № stages	SB	Sex by development interaction	%
**1**	2272	811	35.7
**2**	390	253	64.9
**3**	9	3	33.3

Supplemental table 9| *Timema californicum* samples and their accession numbers

Experiment Accession	Sample Accession	Run Accession	Library Name	Developmental stage and sex	Total Spots	Total Bases
SRX9531643	SRS7738165	SRR13084978	Tcm_M_H_rep1	hatchling_male	44601473	9009497546
SRX9531642	SRS7738164	SRR13084979	Tcm_F_H_rep1	hatchling_female	44379870	8964733740
SRX9531641	SRS7738163	SRR13084980	Tcm_M_A_rep4	adult_male	37276088	7529769776
SRX9531640	SRS7738162	SRR13084981	Tcm_M_A_rep3	adult_male	35119725	7094184450
SRX9531639	SRS7738161	SRR13084982	Tcm_M_A_rep2	adult_male	39800843	8039770286
SRX9531638	SRS7738160	SRR13084983	Tcm_M_A_rep1	adult_male	36436465	7360165930
SRX9531637	SRS7738159	SRR13084984	Tcm_F_A_rep4	adult_female	39841828	8048049256
SRX9531636	SRS7738158	SRR13084985	Tcm_M_J_rep4	juvenile_male	19806861	4000985922
SRX9531635	SRS7738157	SRR13084986	Tcm_F_J_rep3	juvenile_female	43925792	8873009984
SRX9531634	SRS7738156	SRR13084987	Tcm_F_J_rep2	juvenile_female	43805442	8848699284
SRX9531633	SRS7738155	SRR13084988	Tcm_F_J_rep1	juvenile_female	47842363	9664157326

SRX9531632	SRS7738154	SRR13084989	Tcm_F_A_rep3	adult_female	38345072	7745704544
SRX9531631	SRS7738153	SRR13084990	Tcm_M_J_rep3	juvenile_male	41828810	8449419620
SRX9531630	SRS7738152	SRR13084991	Tcm_M_J_rep2	juvenile_male	44470745	8983090490
SRX9531629	SRS7738151	SRR13084992	Tcm_M_J_rep1	juvenile_male	49269834	9952506468
SRX9531628	SRS7738150	SRR13084993	Tcm_F_H_rep4	hatchling_female	45158508	9122018616
SRX9531627	SRS7738149	SRR13084994	Tcm_M_H_rep5	hatchling_male	48849073	9867512746
SRX9531626	SRS7738148	SRR13084995	Tcm_F_H_rep3	hatchling_female	63932274	12914319348
SRX9531625	SRS7738147	SRR13084996	Tcm_M_H_rep4	hatchling_male	46547428	9402580456
SRX9531624	SRS7738146	SRR13084997	Tcm_F_H_rep2	hatchling_female	40703580	8222123160
SRX9531623	SRS7738145	SRR13084998	Tcm_M_H_rep3	hatchling_male	42144046	8513097292
SRX9531622	SRS7738144	SRR13084999	Tcm_M_H_rep2	hatchling_male	37358520	7546421040
SRX9531621	SRS7738143	SRR13085000	Tcm_F_A_rep2	adult_female	36838347	7441346094
SRX9531620	SRS7738142	SRR13085001	Tcm_F_A_rep1	adult_female	38243875	7725262750

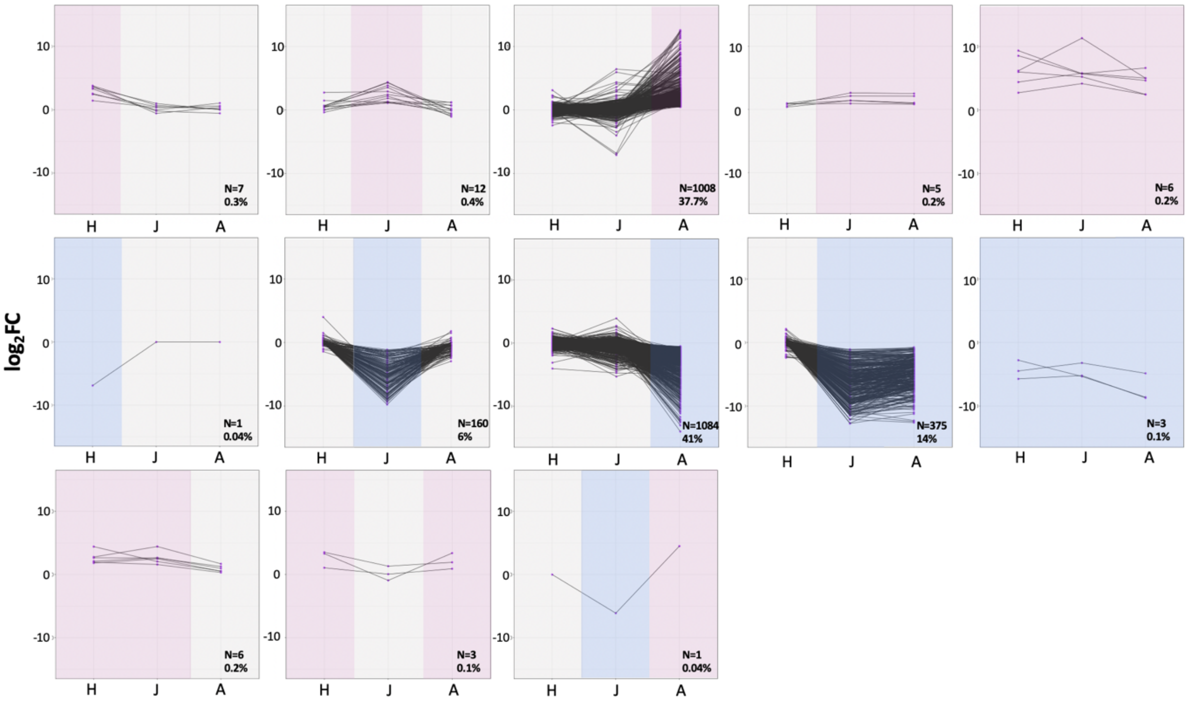

Supplemental figure 1| Categories of sex-biased genes in *T. californicum* ("H"- hatchling, "J"- juvenile, "A"- adult). Blue background stands for male-biased state of a gene, pink background stands for female-biased state of a gene, and gray background stands for un-biased state of a gene. For each category the number of genes (N) and their percentage is marked at the bottom right corner.

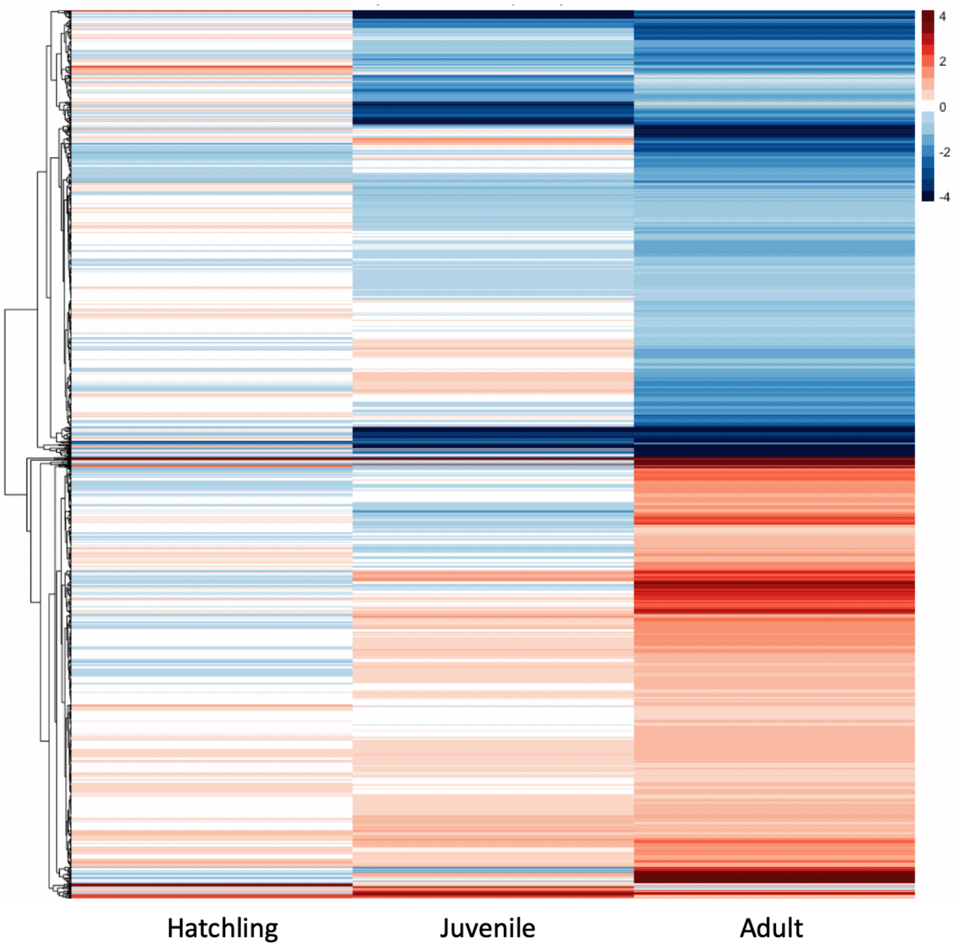

Supplemental figure 2| Heat-map showing the log_2FC over three developmental stages of 1887 genes that were significantly sex-biased in at least one stage. Note that a subset of genes (n=784) was removed due to low expression in some of the stages. Genes in red are female-biased, blue are male-biased.

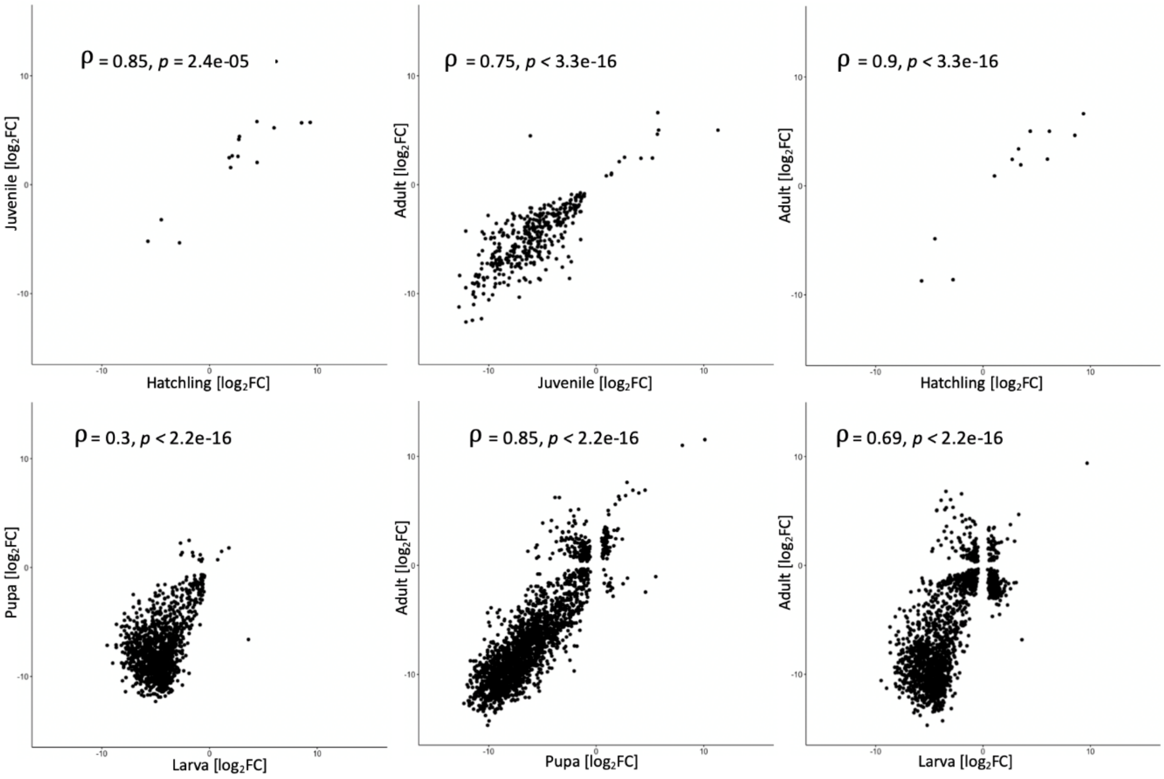

Supplemental figure 3 | Spearman's correlations of sex-biased gene expression [log_2FC] between developmental stages. Top panels: *T. californicum*; bottom panels: *D. melanogaster.* Spearman's correlation coefficients and adjusted p values are shown in each panel.

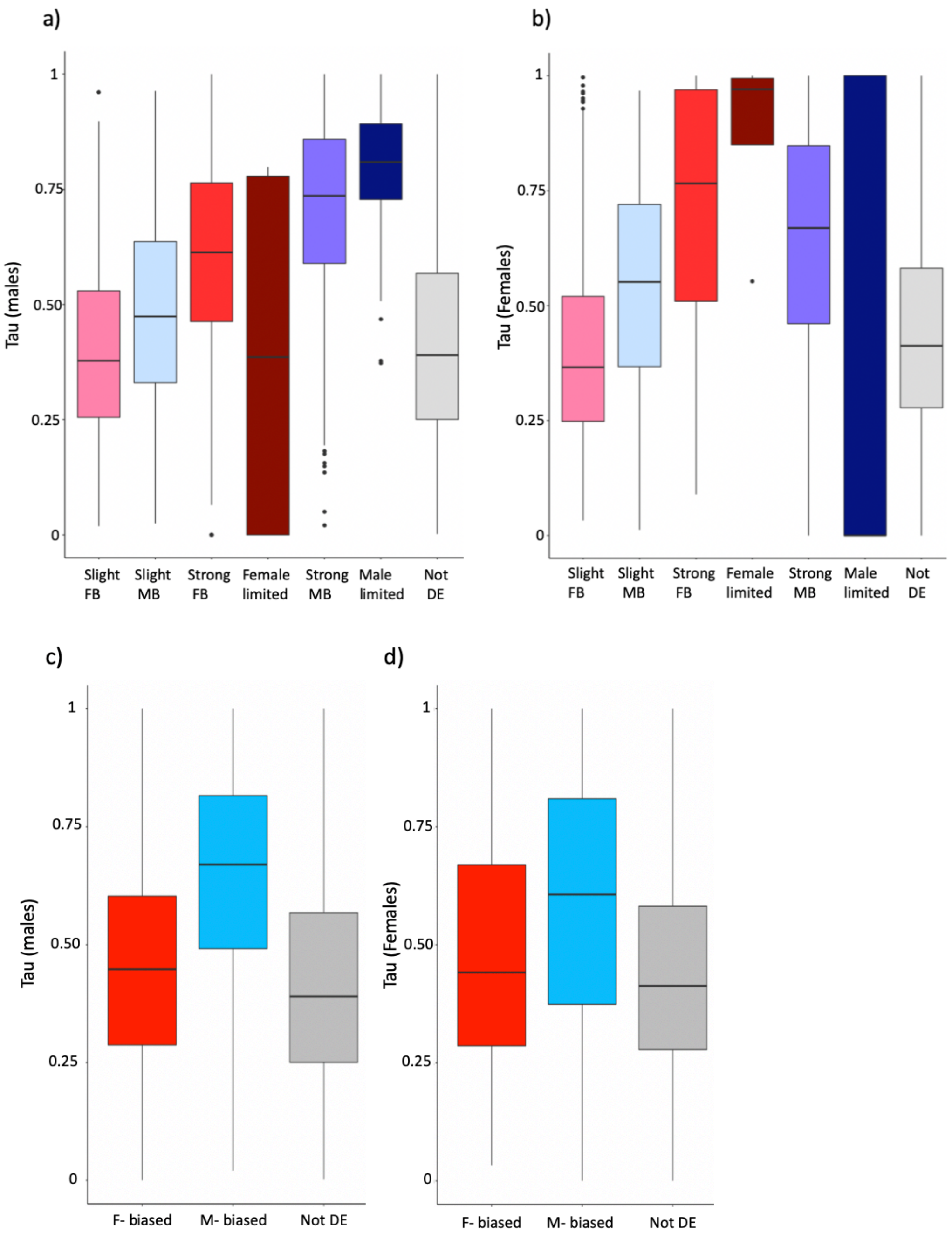

Supplemental figure 4 | Tau index of gene expression in males (a, c) and females (b, d), for each sex-bias category; slight FB, slight MB, strong FB, strong MB, Female-limited, Male limited, Not DE (a,b), and per broader sex-bias categories; F-biased, M-biased and Not DE. Tau ranges from zero (uniformly expressed over development) to one (gene expressed in only one stage). Gene categories are depicted with different colors; boxplots represent the median, lower and upper quartiles, and whiskers the minimum and maximum values (in the limit of 1.5x interquartile range).

a) Hatchlings

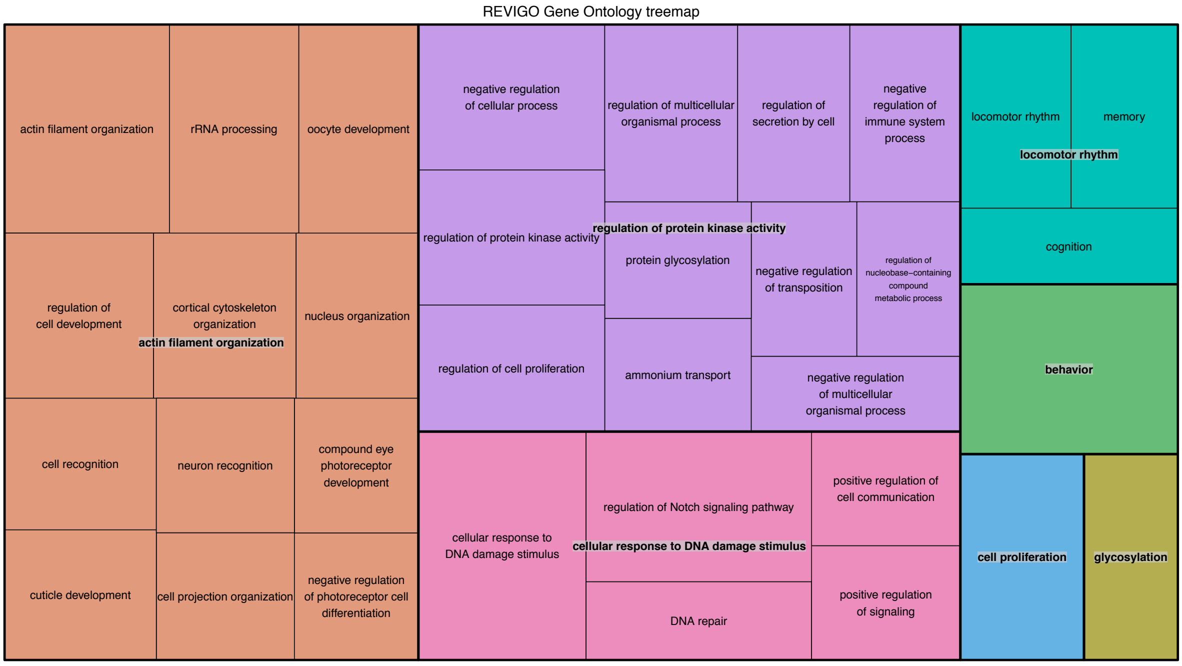

b) Juveniles

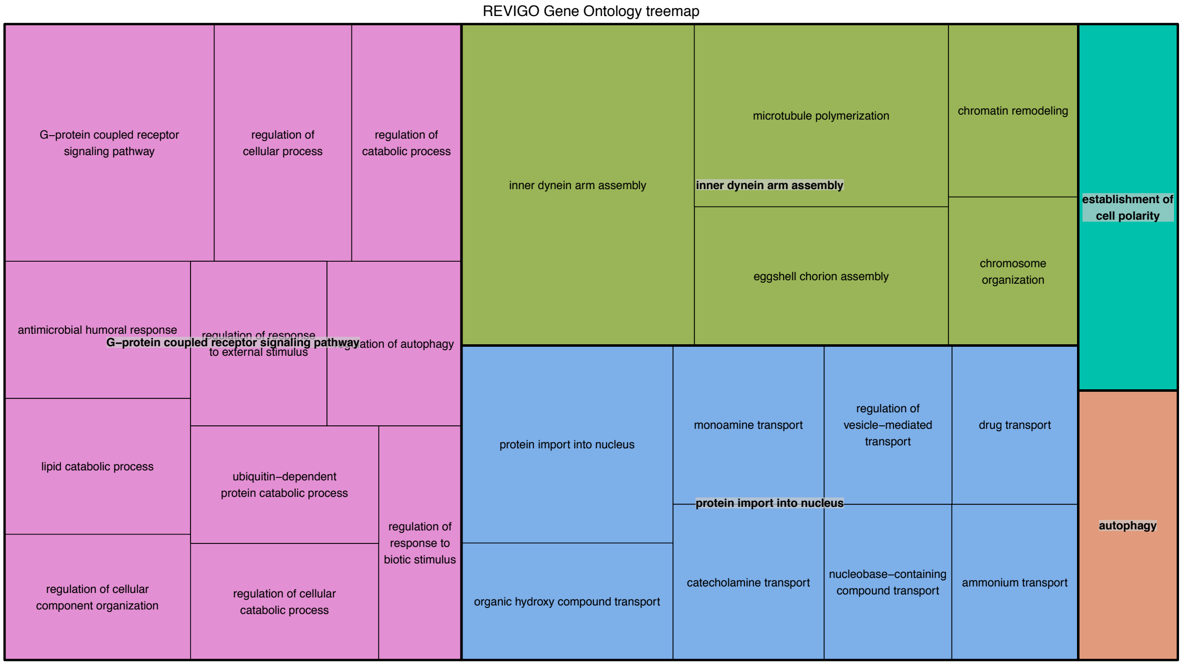

c) Adults

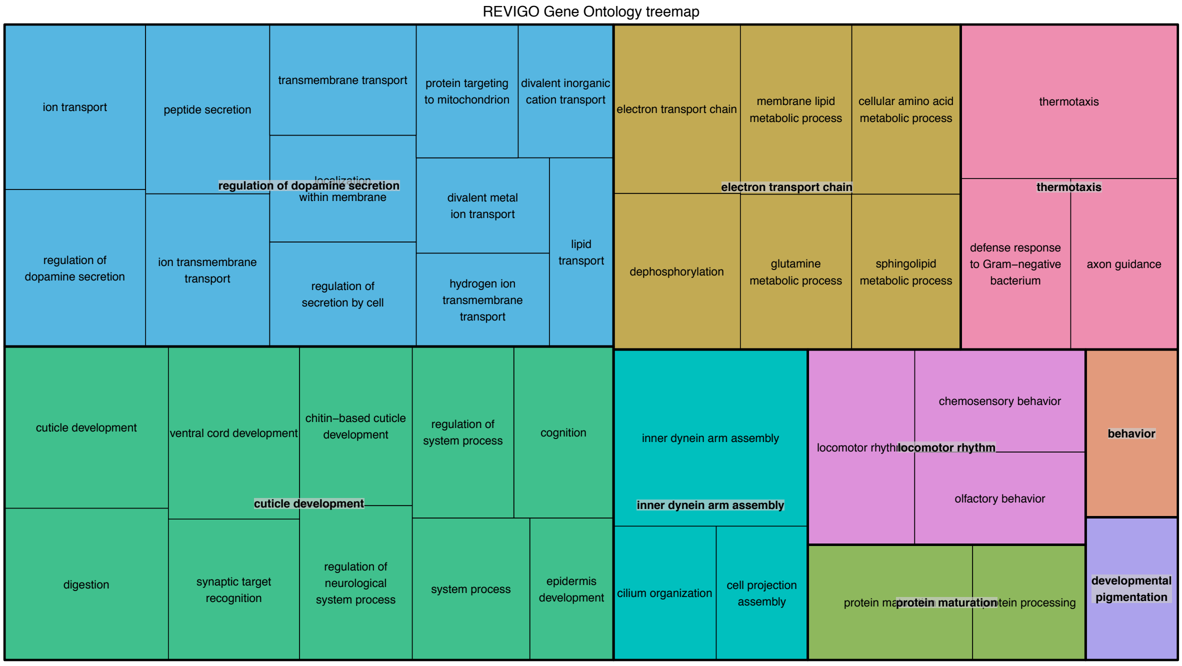

Supplemental figure 5 | Summary of significant GO terms by Revigo (Supek et al., 2011) for genes that are sex-biased in a) hatchlings, b) juveniles and c) adults.

Bibliography

Supek, F., Bosnjak, M., Skunca, N., and Smuc, T. (2011). REVIGO summarizes and visualizes long lists of gene ontology terms. PLoS One *6*, e21800.
